# Comparison of the effect of postweaning social isolation, enriched environment, and exercise training on learning and memory functions in rats

**DOI:** 10.55730/1300-0144.5708

**Published:** 2023-06-21

**Authors:** Hatice EKİNALAN KAYHAN, Nilsel OKUDAN, Muaz BELVİRANLI

**Affiliations:** 1 Department of Radiotherapy, Vocational School of Health Services, Ankara University, Ankara, Turkiye; 2 Department of Physiology, Faculty of Medicine, Selçuk University, Konya, Turkiye

**Keywords:** Social isolation, environment, exercise training, Morris water maze test, brain-derived neurotrophic factor, nerve growth factor

## Abstract

**Background/aim:**

To assess the effects of postweaning social isolation, an enriched environment, and exercise training on learning and memory functions in rats, as well as their relation with the brain-derived neurotrophic factor (BDNF) and nerve growth factor (NGF) concentrations in the hippocampus.

**Materials and methods:**

Randomly assigned into 4 groups were 35 female postweaning rats (25 day old), as the control (C), social isolation (SI), enriched environment (EE), and exercise training (E) groups. The SI and the EE groups were housed under their specific conditions and the E and the C groups were housed under standard conditions for 6 weeks. The rats in the E group swam for 60 min/day, 5 days a week, for 6 weeks. After 6 weeks, the rats were evaluated in the Morris water maze (MWM). Following MWM assessment, hippocampal tissue and blood samples were taken to measure the BDNF and NGF.

**Results:**

According to the results of the MWM probe trial session, the thigmotaxis behavior was higher in the SI group compared to the C group (p < 0,05). Furthermore, the time spent in the target quadrant (quadrant with an escape platform) was lower in the SI group compared to the EE group (p < 0.05). The BDNF and NGF levels in the hippocampus and plasma were not different between the groups (p < 0.05).

**Conclusion:**

Postweaning social isolation may increase thigmotaxis behaviors. Postweaning social isolation, enriched environment, and exercise training did not affect the spatial learning, memory function, hippocampal BDNF or NGF levels in female rats.

## 1. Introduction

The housing environment can cause various behavioral, physiological, and cognitive changes in animals and humans. Social isolation and an enriched environment can be given as examples of different housing environments [1]. Postweaning social isolation causes various behavioral and biochemical changes. It has been stated that social isolation may cause an increase in disordered sensorimotor reactivity, aggression, cognitive rigidity, depression-like behaviors, and anxiety-related behaviors [1–5]. Social isolation also reduces spatial learning, synaptic plasticity, hippocampal neurogenesis, neurotrophic factor expression, long-term potentiation (LTP) in hippocampal tissue and causes histochemical changes in oligodendrocyte maturation and myelinization [1, 2, 6]. On the other hand, an enriched environment creates anatomical and physiological changes in the brain, such as the development of learning and memory functions in spatial tasks, hippocampal neurogenesis, dendritic branching, synaptogenesis, and increased LTP production [7–9]. Different housing environments, such as social isolation and an enriched environment, can affect learning and memory by altering synaptic plasticity and LTP [1, 10].

Exercise training (aerobic and resistance) improves spatial learning and memory functions associated with the hippocampus. This effect of exercise training can be explained by changes in the frequency of synaptic activation and an increase or decrease in the long-term efficiency of the synapses [11]. Studies have shown that both forced (treadmill) and unforced exercise (activity wheel) training increase hippocampal neurogenesis, cell proliferation, and dendritic branching. These effects can be explained by the fact that exercise training modulates the release and utilization of neurotransmitters such as monoamines, neurotrophic factors such as brain-derived neurotrophic factor (BDNF), and growth factors [11, 12].

BDNF and nerve growth factor (NGF) are members of the neurotrophin family. It has functions such as controlling neuronal survival and differentiation in the nervous system, regulation of synaptogenesis, and activity-related synaptic plasticity [13]. BDNF regulates synaptic plasticity in axon collaterals and dendrites, which results in the strengthening or weakening of connections between neurons by using or not using the underlying learning and memory functions of synaptic pathways [13]. NGF plays an important role in the survival, growth, protection, and maintenance of neurons in the peripheral and central nervous system [14]. Postweaning social isolation, an enriched environment, and exercise affect BDNF and NGF expression [6, 11, 15].

Previous studies have generally investigated the effects of exercise training and an enriched environment on rats with various diseases and injuries [2, 14, 16]. This study examined how multienvironment conditions and exercise training affect learning, memory functions, and their behavioral and biochemical dimensions in healthy postweaning rats. The current investigation aimed to compare the effect of postweaning social isolation, an enriched environment, and exercise training on learning and memory functions in rats based on the Morris water maze (MWM) test, as well as on BDNF and NGF levels in hippocampal tissue.

## 2. Materials and methods

### 2.1. Experimental animals and study groups

**Figure 1:**
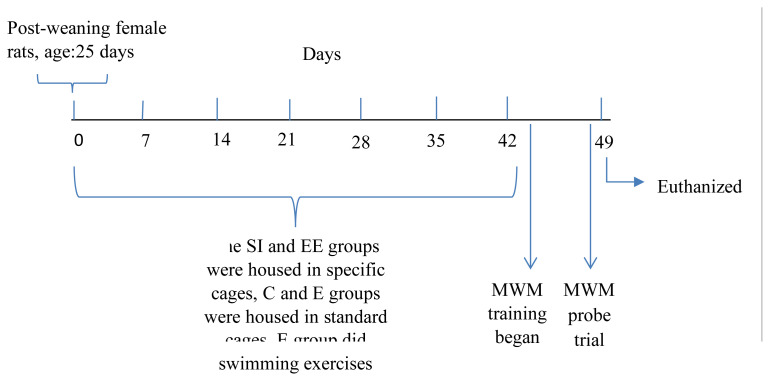
Draft of the study design. SI: Social isolation group, EE: enriched environment group, C: control group, E: exercise group, and MWM: Morris water maze.

The study included postweaning female Wistar rats (n = 35), aged 25 days, and weighing 60–100 g. The rats were housed under a light/dark photoperiod of 12:12, at 21 ± 2 °C, and 50% humidity, with access to food and water ad libitum. After weaning, the rats were randomly assigned into 4 groups, as the control (C) (n = 6), social isolation (SI) (n = 10), enriched environment (EE) (n = 10), and exercise training (E) (n = 9) groups. The ability to use spatial clues, the volumes of the hippocampus and subregions associated with spatial memory functions, and performances in the MWM differ in male and female genders. Therefore, this study focused on a single gender. Female rats were chosen because there are few studies on females and they showed better performance in learning the MWM [17–19]. The study design is demonstrated in Figure 1.

### 2.2. Housing conditions

Each rat in the SI group was housed alone in isolation cages (42 × 27 × 19 cm) for 6 weeks. The isolation cages consisted of cages with a special wooden box. The wooden box had 4 walls and the top was open. The cage was seated in a wooden box. The wooden box was located just outside of the cage. The function of the wooden box was to provide social isolation for 6 weeks in the SI group and reduce communication with the environment. Other visual, auditory, and olfactory conditions were the same for all of the groups, including the SI group. The rats in the SI group were not touched in any way, except when cleaning the cage once a week.

The EE group was housed in large (60 × 38 × 20 cm) enriched cages, as a group of 5, for 6 weeks. The enriched cages included various toys of different colors, shapes, and textures including tunnels, colored balls, ladders, mirrors, platform, and nesting materials, but no running wheels. The toys were removed, washed in alcohol, and interchanged between the cages twice a week.

The E and C groups were housed in standard cages (42 × 27 × 19 cm), as a group of 3–4, for 6 weeks.

### 2.3. Swimming exercise protocol

The swimming exercise was performed in a water tank (155 × 80 × 70 cm), at 25 ± 2 °C, and a depth of 50 cm. The E group performed the swimming exercise for 1 h each day, for 5 weeks. In the familiarization period, the rats were acclimatized to the swimming exercise for 1 week (20 min each day). The swimming exercise was done between 12:00 and 13:00 h.

### 2.4. Morris water maze test

Spatial learning and memory functions were evaluated via the MWM. The MWM consisted of a circular water tank (150 × 60 cm). The water temperature was adjusted to 25 ± 2 °C. The water was colored with nontoxic paint. The maze was divided into 4 imaginary quadrants: southeast (SE), southwest (SW), northeast (NE), and northwest (NW). A square platform (10 × 10 cm), the escape platform, was located in the NW quadrant and was placed 2 cm below the water surface and 10–15 cm away from the tank wall and was fixed throughout the training period. Different a few identical visual cues were placed on the wall of the room for the spatial orientation of the rats. The MWM test was performed between 12:00 and 13:00 h. The experiment was set up as 4 days of the training phases and 1 day of the learning phase. A 4-day training experiment was conducted. Each rat performed 4 trials once a day, starting from a different quadrant each day. After the rat was released from any quadrant into the water, it was given 60 s to find the platform. If the rat could not find the platform within 60 s, it was picked up by hand and placed on the platform, and it was kept there for 30 s to observe the environment and learn its location. During the training trials, 4 parameters were recorded: the latency to find the platform (s), total distance traveled (cm), average swimming speed (cm/s), and thigmotactic behavior (s).

A probe trial or learning phase was performed to evaluate the memory of the rats 24 h after the last training session. In the probe trial, the escape platform was removed from the tank and the rats were allowed to swim freely for 90 s. The following parameters were recorded: total distance traveled, swimming speed, thigmotactic behavior, time spent in each quadrant, time spent in the platform area, number of platform crossings.

### 2.5. Tracking system

MWM test data were recorded with a computerized video monitoring system located above the test area. MWM data were evaluated using special software (Noldus Information Tech. Ethovision, XT 10.0, Wageningen, The Netherlands).

### 2.6. Blood and tissue samples

The rats were anesthetized with ether 24 h after the MWM test, and blood samples were taken quickly. Then the rats were euthanized by cervical dislocation and the hippocampus tissues were dissected. The hippocampus samples were washed with ice-cold saline. They were frozen rapidly with liquid nitrogen and stored at −80 °C until use for the biochemical analyses.

The hippocampus samples were measured with precision (0.001 g) scales (Sartorius, M-power, Germany) and the net weight was calculated. They were homogenized with phosphate buffer (Wise Mix HG-15; Daihan Scientific, Seoul, Korea, Ph 7.4). Homogenization was performed on ice with an ultrasonic tissue homogenizer. The tissues were homogenized at 3000 rpm and 4 °C, followed by centrifugation for 30 min (1200 NF Core, Turkey) and the supernatants were used for the analyses.

The hippocampus and plasma BDNF levels were measured using a rat BDNF enzyme-linked immunosorbent assay (ELISA) kit (Sunred Biological Technology, Cat. No. 201-11-0477, Shanghai, China) and the ELISA reader (Powerwave XS, Biotek, USA) according to the manufacturer’s instructions. The analytical sensitivity of the kit was 0.035 ng/mL and the detection limit was 0.04–10 ng/mL.

The hippocampus and plasma NGF levels were measured using a rat NGF ELISA kit (Sunred Biological Technology) and the ELISA reader (Biotek) according to the manufacturer’s instructions. The analytical sensitivity of the kit was 0.276 ng/mL and the detection limit was 0.3–90 ng/mL.

### 2.7. Statistical analyses

Statistical analyses were performed using IBM SPSS Statistics for Windows 22.0 (IBM Corp., Armonk, NY, USA). The results were presented as the mean ± SD. The suitability of the variables to normal distribution was evaluated using the Shapiro-Wilk test. The normally distributed parameters were analyzed using 1-way analysis of variance (ANOVA). The post hoc Tukey HSD test was used to determine the differences between the groups. Data that were not normally distributed were analyzed using the Kruskal-Wallis test. The data recorded in the MWM training sessions were evaluated by repeated measures ANOVA. Statistical significance was accepted as p < 0.05.

## 3. Results

**Figure 2:**
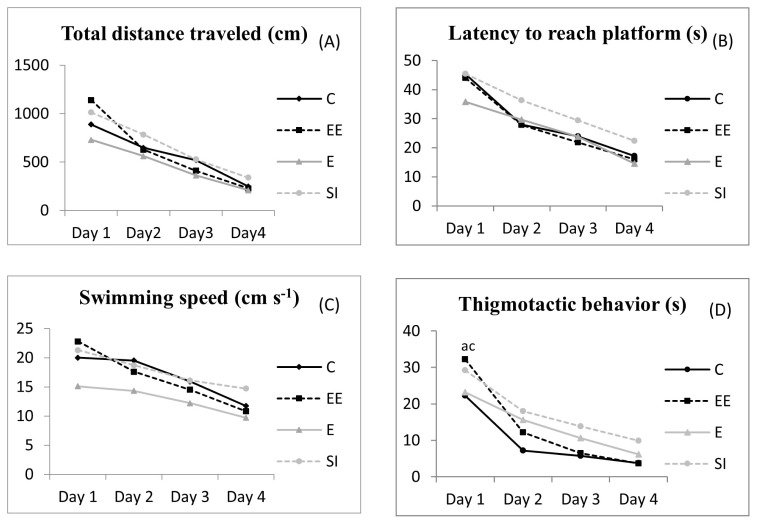
(A) Total distance traveled, (B) latency to reach the platform, (C) average swimming speed, and (D) thigmotactic behaviors during the MWM training sessions.

The total distance traveled, latency to reach the platform, average swimming speed, and thigmotactic behavior significantly decreased in all of the groups with the consecutively repeated trials (p < 0.05) (Figures 2A–2D). This result was valuable, as it was a sign that the rats learned the task and were ready for the MWM probe session.

**Figure 3:**
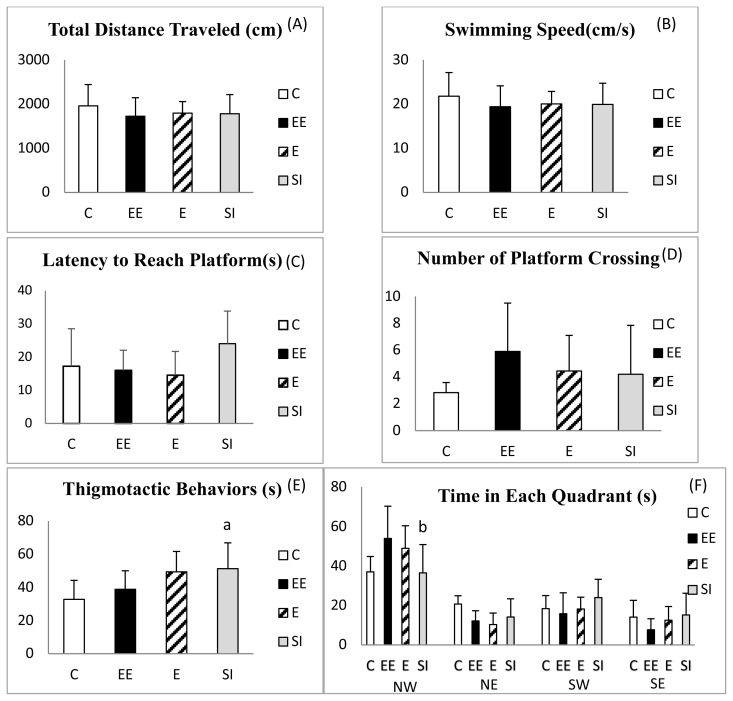
(A) Total distance traveled, (B) average swimming speed, (C) latency to reach the platform, (D) number of platform crossings, (E) thigmotactic behaviors, and (F) time in each quadrant during the MWM probe sessions. Variables are shown as the mean ± SD. ^a^p < 0.05 compared to the C group and ^b^p < 0.05 compared to the EE group.

Figure 3A shows the total distance traveled, 3B shows the average swimming speed (cm/s), 3C shows the latency to the reach platform, 3D shows the number of platform crossings, 3F shows the thigmotactic behaviors, and 3E shows the time in each quadrant of the groups during the MWM probe session. In the MWM probe session, there was no significant difference between the groups in terms of the total distance traveled, average swimming speed, number of platform crossings, and latency to reach the platform (p > 0.05). However, the thigmotactic behaviors were higher in the SI group than in the C group (F = 3.7737 and p = 0.020) (Figure 3E). In addition, the time spent in the target quadrant (quadrant with an escape platform) was lower in the SI group than in the EE group (F = 3.768 and p = 0.020) (Figure 3F).

The Table shows the hippocampus and plasma BDNF and NGF levels, which were not different between the groups (p > 0.05).

## 4. Discussion

The present study examined a comparison of the effects of postweaning social isolation, an enriched environment, and exercise training on the learning and memory functions in rats, as well as BDNF and NGF levels in hippocampal tissue.The MWM is one of the most popular behavioral tests that has been used for assessing the spatial learning and memory of rodents. The MWM also provides information about thigmotactic behaviors that occur in stressful situations [20, 21]. Thigmotactic behavior refers to the tendency of an animal to move along the edges around it [22, 23]. It is generally considered to be an indicator of anxiety or fear and has been reported to be associated with high corticosteroid levels. When thigmotactic behavior occurs, the discovery process of the animal is disrupted, it becomes difficult for them to find the target, and the time to reach the target is prolonged [22, 23]. It has been stated that social isolation stress in early life that causes thigmotactic behavior can affect brain development and neuroplasticity, cause behavioral changes and motor skill deficiencies, and impair spatial learning and memory. Numerous studies have reported that social isolation increases anxiety and aggression, causes hyper locomotion, and is associated with decreased memory ability [2, 5, 24–31]. In addition, social isolation was found to be associated with an increase in anxiety-related behaviors in the open field and elevated plus maze tests [32, 33]. In the current study, the thigmotactic behavior during the MWM probe session was significantly higher in the SI group than in the C group. While social isolation caused a change in the behavioral dimensions of neuroplasticity, it did not change in the biochemical dimension.

Numerous studies have demonstrated the impact of an enriched environment on brain plasticity, morphology, learning, and memory functions [34–37]. An enriched environment includes cognitive, social, and physical different stimulating factors. These stimulations can improve learning and brain function in different ways. An enriched environment improves neuroplasticity due to changes in protein synthesis, and synaptic and cell morphology. Many studies have reported that exposure to an enriched environment increases the expression of neurotrophic factors, primarily NGF and BDNF [8,38]. According to Kobilo et al. and Mustroph et al., exercise is the main stimulating factor for increased neurogenesis and BDNF expression in the brain, and they found that enrichment alone had no effect on these mechanisms [39, 40] On the contrary, Birch et al. reported that an enriched environment has a time-dependent cognitive enhancing effect independent of physical activity [41]. Herein, no running wheels were placed in the cages, so as to examine the pure effect of the enriched environment in the study. Although the time spent in the target quadrant in the MWM probe session was higher in the EE group than in the SI group, the plasma and hippocampus BDNF and NGF levels were not different than those in the other groups. These studies, combined with the new data presented herein, highlight the complex nature of the mechanisms underlying enrichment-induced memory improvements.

Exercise can enhance various forms of learning in young rats, such as 8-arm radial maze navigation [42], passive avoidance [43], and fear conditioning [44]. Exercise-induced improvements may not be observed consistently [45, 46]. This is because routine tests such as the MWM are used to assess learning and the ceiling effect is evident in the test. The fact that some tasks are optimally learned by young healthy rats, leaving no capacity to promote exercise-related improvements, may mask the cognitive enhancing properties of exercise. Evidence from the literature suggests that exercise more reliably improves cognitive performance in the presence of an existing deficit [11, 47]. The fact that healthy rats were used in the current study and that there was no significant difference in the MWM test results in the E group compared to the other groups supports this information in the literature.

Many studies have reported that exercise increases the expression of neurotrophic factors in the hippocampus, improves neural survival, differentiation, connectivity, and plasticity, and has a protective effect against depression and chronic stress [48–50]. It was reported that after acute treadmill running [51], voluntary exercise [52], and chronic voluntary exercise after traumatic brain injury [53], the BDNF levels in the hippocampus increased. It was also stated that the forced exercise used in the studies, when combined with stressors such as electric shock during treadmill exercise or putting the rats in water, can increase corticosterone levels and reduce the beneficial effect of exercise on neuron structure and function [47, 51, 54]. In the present study, group E performed the swimming exercise for 1 h a day. There was no significant difference in the plasma and hippocampus BDNF and NGF levels in group E compared to the other groups. A possible reason for not observing the positive effects of exercise on learning in the BNDF and NGF levels may be that swimming exercise creates stress in rats and reduces the positive effects of exercise.

One of the limitations of this study was that only the BDNF and NGF protein levels were examined, but not gene expression. Another limitation was that the study was conducted only on female rats and no other test evaluating anxiety, stress, and thigmotactic behaviors was used. Additionally, the corticosterone levels of the rats during the swimming exercise and social isolation were not measured, because sometimes, even enrichment may be stressful and increase corticosterone levels.

In conclusion, postweaning social isolation increased the thigmotactic behaviors in female rats. Postweaning social isolation, an enriched environment, and exercise training did not alter the spatial learning and memory functions in female rats in the MWM test. Moreover, the BDNF and NGF levels of the hippocampus and plasma did not change. Additional studies are needed to examine the effects of housing conditions and exercise on learning and memory functions and detail the relationship between BDNF and NGF levels in female rats.

## Figures and Tables

**Table:** Hippocampus and plasma BDNF and NGF levels (mean ± SD).

	C group	EE group	E Group	SI Group
BDNF-P (ng/mL)	1.7 ± 0.2	1.5 ± 0.2	1.6 ± 0.2	1.5 ± 0.3
NGF-P (ng/mL)	2.0 ± 0.6	1.7 ± 0.4	1.6 ± 0.2	2.0 ± 0.7
BDNF-H (ng/mg of protein)	3.7 ± 2.5	3.1 ± 2.2	3.6 ± 2.6	1.7 ± 0.8
NGF-H (ng/mg of protein)	3.2 ± 1.9	3.1 ± 2.2	2.7 ± 1.8	1.6 ± 0.7

Brain-derived neurotrophic factor plasma level (BDNF-P), nerve growth factor plasma level (NGF-P), BDNF hippocampus level (BDNF-H), and NGF hippocampus level (NGF-H).
